# Individual chitin synthase enzymes synthesize microfibrils of differing structure at specific locations in the *Candida albicans* cell wall

**DOI:** 10.1111/j.1365-2958.2007.05990.x

**Published:** 2007-12

**Authors:** Megan D Lenardon, Rhian K Whitton, Carol A Munro, Deborah Marshall, Neil A R Gow

**Affiliations:** 1 School of Medical Sciences, University of Aberdeen, Institute of Medical SciencesForesterhill, Aberdeen AB25 2ZD, UK.; 2 Department of Medical Microbiology, University of Aberdeen, Polworth BuildingForesterhill, Aberdeen AB25 2ZD, UK.

## Abstract

The shape and integrity of fungal cells is dependent on the skeletal polysaccharides in their cell walls of which β(1,3)-glucan and chitin are of principle importance. The human pathogenic fungus *Candida albicans* has four genes, *CHS1*, *CHS2*, *CHS3* and *CHS8*, which encode chitin synthase isoenzymes with different biochemical properties and physiological functions. Analysis of the morphology of chitin in cell wall ghosts revealed two distinct forms of chitin microfibrils: short microcrystalline rodlets that comprised the bulk of the cell wall; and a network of longer interlaced microfibrils in the bud scars and primary septa. Analysis of chitin ghosts of *chs* mutant strains by shadow-cast transmission electron microscopy showed that the long-chitin microfibrils were absent in *chs8* mutants and the short-chitin rodlets were absent in *chs3* mutants. The inferred site of chitin microfibril synthesis of these Chs enzymes was corroborated by their localization determined in Chsp–YFP-expressing strains. These results suggest that Chs8p synthesizes the long-chitin microfibrils, and Chs3p synthesizes the short-chitin rodlets at the same cellular location. Therefore the architecture of the chitin skeleton of *C. albicans* is shaped by the action of more than one chitin synthase at the site of cell wall synthesis.

## Introduction

*Candia albicans* is a dimorphic human pathogenic fungus capable of causing systemic infection in an immunocompromised host. Glucan and chitin are the key structural polysaccharides of the *C. albicans* cell wall that provide strength, rigidity and shape and provide a skeletal framework onto which cell wall mannoproteins are linked (Chaffin *et al*., 1998; Kapteyn *et al*., 2000; Klis *et al*., 2001; Munro and Gow, 2001; Roncero, 2002). Although chitin comprises only a minor component of the *C. albicans* cell wall (Klis *et al*., 2001), chitin synthesis is essential for viability (Shaw *et al*., 1991; Munro *et al*., 2001).

The enzymes involved in fungal cell wall biosynthesis lack human homologues. Echinocandins which target β(1,3)-glucan synthesis are the only antifungal drugs in current use that target the cell wall (Onishi *et al*., 2000; Denning, 2003; Odds *et al*., 2003), and chitin synthesis remains a potential but as yet unrealized target for future antifungal chemotherapy (Bowman and Free, 2006). Most fungi have several chitin synthase enzymes whose co-ordinated activity synthesizes chitin at various sites in the cell wall at specific stages in the cell cycle (Munro and Gow, 2001; Roncero, 2002). Considerable efforts have been made to investigate the individual and collective roles of these gene products; however, the biosynthesis of chitin, the world's second most abundant biopolymer, remains poorly understood at the biochemical and molecular level.

In *C. albicans*, chitin synthesis is achieved by a family of four chitin synthase enzymes encoded by *CHS1*, *CHS2*, *CHS3* and *CHS8* (Gow *et al*., 1994; Bulawa *et al*., 1995; Mio *et al*., 1996; Munro *et al*., 2001; 2003). Analysis of the phenotypes of individual and multiple *chs* mutants in *C. albicans* has given us clues about the functions of each isoenzyme. Chs1p is a class II chitin synthase and is the only chitin synthase reported to date to be essential for growth and viability (Munro *et al*., 2001). The phenotype of a conditional *chs1*Δ null mutant strain grown under restrictive conditions suggests that Chs1p is involved in the synthesis of chitin in the primary septum, and that it also contributes to the integrity of the lateral cell wall (Munro *et al*., 2001). Chs2p and Chs8p are both class I chitin synthase enzymes. Chs2p represents the major chitin synthase activity measured in cell membrane preparations (Gow *et al*., 1994). Chs8p makes a minor contribution to chitin synthase activity measured *in vitro*, but the biological significance of Chs8p is unknown (Munro *et al*., 2003). Chs3p is a class IV chitin synthase and is responsible for the synthesis of the majority of chitin in the cell wall of yeast and hyphal cells (Bulawa *et al*., 1995; Mio *et al*., 1996; Munro *et al*., 1998).

Although chitin is a chemically homogeneous molecule comprising a homopolymer of β(1,4)-*N*-acetylglucosamine (GlcNAc), it can exist in a number of distinct morphological forms (Rudall, 1963). Fungal cell walls consist of α-chitin, where adjacent chains are antiparallel and bridged by strong interchain hydrogen bonds (Minke and Blackwell, 1978). Other organisms have β-chitin arranged in parallel chains or have mixtures of α- and β-chitin (Rudall, 1963; Gooday and Schofield, 1995). Chitin synthases are located in the plasma membrane with their active site on the cytoplasmic face (Nagahashi *et al*., 1995). The substrate of chitin, uridine diphosphate-*N*-acetylglucosamine (UDP-GlcNAc), is present in the cytoplasm, and it is presumed that nascent chitin chains are extruded through the Chs protein or plasma membrane into the extracellular space before the individual chitin chains come together, fold back on themselves and hydrogen bond to form chitin microfibrils (Herth, 1980; Elorza *et al*., 1983; Cabib *et al*., 1988; Bulawa, 1993; Munro and Gow, 2001).

Nascent chitin microfibrils are thought to undergo a gradual process of maturation to a more robust, inert form as immediately after its synthesis, chitin is sensitive to chitinase but becomes more resistant over time (Vermeulen and Wessels, 1984; 1986; Gooday and Schofield, 1995). As the cell wall matures, there is evidence that chitin becomes covalently attached to β(1,3)- and β(1,6)-glucan, which, in turn, is attached to various cell wall proteins (Hartland *et al*., 1994; Kollar *et al*., 1995; 1997). Such cross-linking may also affect susceptibility to chitinase.

The robust nature of chitin facilitates extraction and purification methods based on treatments with acid and alkali, which solubilize glucans and other cell wall components (Hunsley and Burnett, 1968). Chitin purified in this way has been shown to be free of glucan contamination, but to retain its native α-conformation as shown by infrared and X-ray crystallography (Gow *et al*., 1980; Gow and Gooday, 1987). The ultrastructure of chitin in various fungi has been studied by shadow-cast transmission electron microscopy (TEM) (Hunsley and Burnett, 1968; Carbonell *et al*., 1970; Gow *et al*., 1980; Gow and Gooday, 1983; Pollack *et al*., 1983; Vermeulen and Wessels, 1984). The architecture of chitin microfibrils imaged in the TEM vary in length, thickness, arrangement and orientation in different fungi, and different structures can exist in a single fungus (Hunsley and Burnett, 1968; Carbonell *et al*., 1970; Gow *et al*., 1980; Gow and Gooday, 1983). In *C. albicans*, chitin in the cell walls of wild-type yeast and hyphal cells were reported to be comprised of short microcrystalline rodlets, while bud scars and septa had longer microfibrils that were interlaced into a chitin network (Gow *et al*., 1980; Gow and Gooday, 1983). The rodlet form of chitin is prevalent in yeast-like cells and dimorphic fungi while the networks of longer microfibrils are common in mycelial fungi (Gow and Gooday, 1983).

Using a panel of isogenic mutant strains that lacked single or multiple chitin synthase genes, we sought to investigate the genetic basis of chitin morphology in *C. albicans*. We show that the assembly of the longer chitin microfibrils is absolutely dependent on the presence of *CHS8*, and that the presence of the short-chitin rodlets found in the cell wall and septa is dependent on *CHS3*. The cellular localization of Chs8p–YFP and Chs3p–YFP in yeast and hyphal cells was consistent with the sites of action of these isoenzymes that were inferred by shadow-cast preparations of chitin ghosts examined by TEM. These results suggest that Chs8p is required for the synthesis of the long-chitin microfibrils in the septum, and Chs3p is required for the synthesis of the short-chitin rodlets in the cell wall and septum, and that these two enzymes determine the overall architecture of the chitin skeleton in the cell wall.

## Results

### Chitin microfibrillar architecture in yeast and hyphal cells

Previous studies have revealed that both long-chitin microfibrils and shorter-chitin rodlets are present in the cell wall and septa of *C. albicans* cells (Gow *et al*., 1980; Gow and Gooday, 1983). To determine the genetic basis of chitin microfibrillar architecture, and to further examine the role of each of the four Chs proteins in the synthesis of chitin at specific locations in the cell, the architecture of chitin microfibrils was examined by shadow-cast TEM of chitin ghosts prepared from wild-type (CAF2-1) and isogenic *chs* mutant strains (Table 1).

**Table 1:** *Candida albicans* strains used and constructed in this study.

Strain name	Strain number	Genotype	Reference
CAF2-1		*URA3*/*ura3*Δ::*imm434*	Fonzi and Irwin (1993)
*chs1*Δ	KWC340	*ura3*Δ::*imm434*/*ura3*Δ::*imm434*, *chs1*Δ::*hisG*/*chs1*Δ::pSK-*URA3-MRP1*p-*CHS1*	Munro *et al*. (2001)
*chs2*Δ	C154	*ura3*Δ::*imm434*/*ura3*Δ::*imm434*, *chs2*::*hisG*/*chs2*::*hisG*-*URA3-hisG*	Mio *et al*. (1996)
chs3Δ	myco3 (C57)	*ura3*Δ::*imm434*/*ura3*Δ::*imm434*, *chs3-2*::*hisG*/*chs3-3*::*hisG-URA3-hisG*	Bulawa *et al*. (1995)
*chs8*Δ	NGY126	*ura3*Δ::*imm434*/*ura3*Δ::*imm434*, *chs8*Δ::*hisG*/*chs8*Δ::*hisG-URA3-hisG*	Munro *et al*. (2003)
*chs1*Δ*chs3*Δ	KWC359	*ura3*Δ::*imm434*/*ura3*Δ::*imm434*, *chs1*Δ::*hisG*/*chs1*Δ::pSK-*URA3-MRP1*p-*CHS1*, *chs3*Δ::*hisG*/*chs3*Δ::*hisG-URA3-hisG*	Munro *et al*. (2001)
*chs2*Δ*chs3*Δ	C156	*ura3*Δ::*imm434*/*ura3*Δ::*imm434*, *chs2*::*hisG*/*chs2*::*hisG*, *chs3*::*hisG*/*chs3*::*hisG-URA3-hisG*	Mio *et al*. (1996)
*chs2*Δ*chs8*Δ	NGY137	*ura3*Δ::*imm434*/*ura3*Δ::*imm434*, *chs2*::*hisG*/*chs2*::*hisG*, *chs8*Δ::*hisG*/*chs8*Δ::*hisG-URA3-hisG*	Munro *et al*. (2003)
BWP17		*ura3*::*imm434*/*ura3*::*imm434*, *his1*::*hisG*/*his1*::*hisG*, *arg4*::*hisG*/*arg4*::*hisG*	Wilson *et al*. (1999)
*CHS1–YFP*	NGY475	*ura3*::*imm434*/*ura3*::*imm434*, *his1*::*hisG*/*his1*::*hisG*, *arg4*::*hisG*/*arg4*::*hisG*, *CHS1*/*CHS1–YFP*:*URA3*, *RPS1*/*RPS1*::CIp30	This study
*CHS2–YFP*	NGY476	*ura3*::*imm434*/*ura3*::*imm434*, *his1*::*hisG*/*his1*::*hisG*, *arg4*::*hisG*/*arg4*::*hisG*, *CHS2*/*CHS2–YFP*:*URA3*, *RPS1*/*RPS1*::CIp30	This study
*CHS3–YFP*	NGY477	*ura3*::*imm434*/*ura3*::*imm434*, *his1*::*hisG*/*his1*::*hisG*, *arg4*::*hisG*/*arg4*::*hisG*, *CHS3*/*CHS3–YFP*:*URA3*, *RPS1*/*RPS1*::CIp30	This study
*CHS8–YFP*	NGY478	*ura3*::*imm434*/*ura3*::*imm434*, *his1*::*hisG*/*his1*::*hisG*, *arg4*::*hisG*/*arg4*::*hisG*, *CHS8*/*CHS8–YFP*:*URA3*, *RPS1*/*RPS1*::CIp30	This study
*CHS1*/*chs1*	NGY20	*ura3*::*imm434*/*ura3*::*imm434*, *CHS1*/*chs1*::*hisG*	Munro (1997)
*CHS2*/*chs2*	NGY479	*ura3*::*imm434*/*ura3*::*imm434*, *his1*::*hisG*/*his1*::*hisG*, *arg4*::*hisG*/*arg4*::*hisG*, *CHS2*/*chs2*Δ*0*::*CdHIS1*	This study
*CHS1–YFP*/*chs1*	NGY480	*ura3*::*imm434*/*ura3*::*imm434*, *CHS1–YFP*:*URA3*/*chs1*::*hisG*	This study
*CHS2–YFP*/*chs2*	NGY481	*ura3::imm434*/*ura3::imm434, his1::hisG*/*his1::hisG, arg4::hisG*/*arg4::hisG,CHS2–YFP:URA3*/*chs2*Δ*0::CdHIS1*	This study

**Fig. 1:**
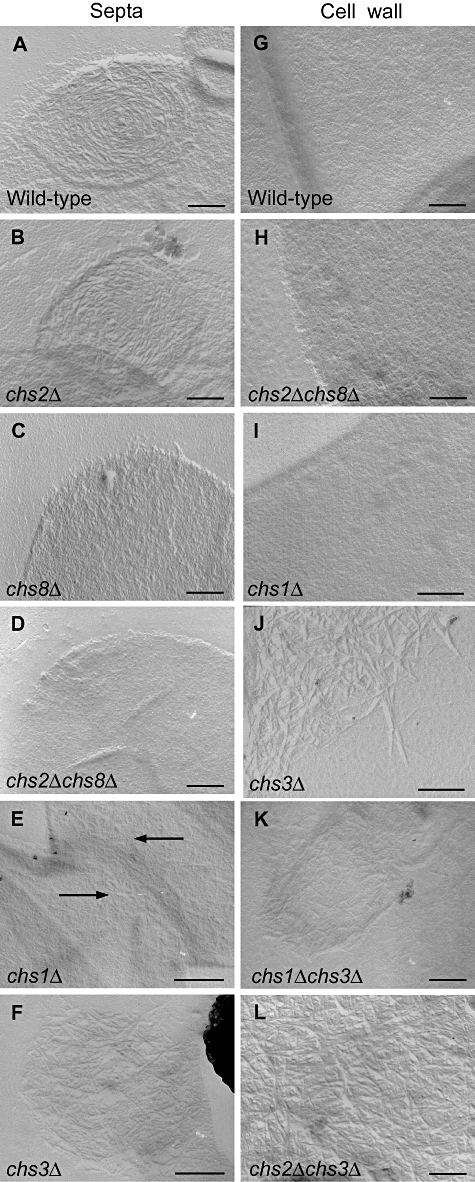
Long-chitin microfibrils require *CHS8* and short-chitin rodlets require *CHS3*. Chitin ghosts from yeast cells grown for 6 h at 30°C in YPD (A–E, G, H, K and L) and hyphal cells grown for 6 h at 37°C in 20% (v/v) FCS (F, I and J) were visualized by shadow-cast TEM. Chitin ghosts from wild-type CAF2-1 (A and G), *chs2*Δ (B), *chs8*Δ (C), *chs2*Δ*chs8*Δ (D and H), *chs1*Δ (E and I), *chs3*Δ (F and J), *chs1*Δ*chs3*Δ (K) and *chs2*Δ*chs3*Δ (L) cells are shown. Long-chitin microfibrils are clearly visible in the bud scars and septal plates of the wild type, *chs2*Δ mutant, *chs3*Δ mutant and at the mother–bud neck region of the *chs1*Δ mutant strain (arrows), but are absent in the bud scars and septa of the *chs8*Δ and *chs2*Δ*chs8*Δ mutant strains, where short-crystalline-chitin rodlets are revealed. Short-chitin rodlets seen clearly in cell walls of the wild-type, *chs2*Δ*chs8*Δ and *chs1*Δ strains are absent in the *chs3*Δ, *chs1*Δ*chs3*Δ and *chs2*Δ*chs3*Δ strains, where long-chitin microfibrils are observed. The scale bars represent 0.5 μm.

Long-chitin microfibrils were visible predominantly in the bud scars and septal plates of wild-type yeast and hyphal cells (Fig. 1A and Fig. S1A). No difference was seen in chitin morphology when *CHS2* was deleted (Fig. 1B and Fig. S1A). The network of long-chitin microfibrils was absent in the bud scars of yeast cells and septal plates of hyphal cells prepared from the *chs8*Δ and *chs2*Δ*chs8*Δ mutant strains (Fig. 1C and D and Fig. S1A). Instead, short-chitin rodlets were revealed in these mutants. The *chs1*Δ conditional mutant strain did not form septa when grown under restrictive conditions; however, long microfibrils were visible either side of the mother–bud neck in yeast cells (Fig. 1E). Long-chitin microfibrils were clearly visible in the septal plates of hyphae prepared from the *chs3*Δ strain (Fig. 1F). Therefore Chs8p is required for the synthesis of the long-chitin microfibrils that are normally present in shadow-cast preparations of the bud scars of yeast cells and septal plates of hyphal cells.

Short-chitin rodlets comprised the bulk of the chitin in cell walls of wild-type yeast and hyphal cells (Fig. 1G and Fig. S1B). The chitin architecture of cell walls prepared from the *chs2*Δ, *chs8*Δ, *chs2*Δ*chs8*Δ and *chs1*Δ mutant strains looked much the same as that in wild-type cells (Fig. S1B, Fig. 1H and I). However, long-chitin microfibrils were present in cell walls prepared from the *chs3*Δ mutant strain (Fig. 1J and Fig. S1B) which were even more prominent in the walls of the *chs1*Δ*chs3*Δ (Fig. 1K) and *chs2*Δ*chs3*Δ mutant strains (Fig. 1L). Therefore Chs3p is required for the synthesis of the short-chitin rodlets in the cell wall. These results suggest either that the cell wall is comprised of short and long microfibrils synthesized by Chs3p and Chs8p, or that Chs enzymes cooperate to synthesize chitin microfibrils of different architectures.

**Fig. 2:**
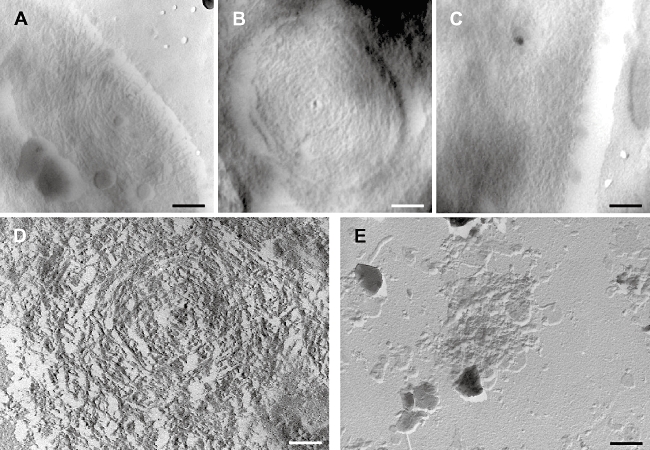
Chitin architecture revealed by enzymic digestion of the cell wall. Chitin ghosts were prepared from wild-type strain CAF2-1 and digested with β(1,6)-glucanase and Quantazyme *ylg*™[β(1,3)-glucanase] (A–C), chitosanase (D) and chitinase (E). The resulting chitin architecture was visualized by shadow-cast TEM. Treatment with β(1,6)-glucanase and Quantazyme *ylg*™ revealed the presence of long-chitin microfibrils at the septum of a hyphal cell (A) and the bud scar of a yeast cell (B), and short-chitin rodlets in the cell wall (C). Limited degradation of chitin in the septa of wild-type cells was observed after treatment with chitosanase (D), compared with the almost complete degradation after treatment with chitinase (E). The scale bars represent 0.5 μm.

To ensure that the observed chitin architecture was not an artefact of the chemical methods used to remove the outer cell wall material, chitinous material was also prepared from wild-type cells by enzymic dissection. After digestion with β(1,3)- and β(1,6)-glucanases, long microfibrils were clearly visible in the septum of hyphae (Fig. 2A) and bud scars of yeast cells (Fig. 2B). Short, rodlet-like microfibrils were visible in the cell wall (Fig. 2C). Therefore chemical extraction did not markedly affect the chitin microfibrillar architecture. We conclude that the alterations in chitin microfibrillar architecture in the *chs* mutant strains were due to the disruption of the *CHS* genes in the mutant strains. To confirm that the cell wall ghosts that were imaged in the TEM were indeed comprised of chitin, preparations from wild-type yeast cells were digested with chitosanase and chitinase. Very slight degradation of the short-chitin rodlets was observed after treatment with chitosanase (Fig. 2D), as can be seen by the fringing of the microfibrils at the edge of the septal plate where it meets the lateral cell wall. This may have been be due to small amounts of chitinase present in the chitosanase preparation. In contrast, almost complete degradation of both the long- and short-chitin microfibrils was observed after the chitinous material was treated with a recombinant source of chitinase (Fig. 2E). Taken together, these control experiments allowed us to conclude that the ghosts that were imaged in the TEM represent chitin in its native conformation and architecture.

### YFP tagging of the Chs proteins

Examination of the chitin microfibrils present in various *chs* mutant strains provided evidence that Chs8p is required for the synthesis of long-chitin fibrils and Chs3p synthesizes short-chitin fibrils. To further examine the location of the chitin synthases in the cell, and to gain insight into how each Chs protein participates in the synthesis of chitin at specific locations in the cell, one copy of each *CHS* gene was tagged with yellow fluorescent protein (*YFP*) at its 3′-end in the strain BWP17 (Table 1) such that the YFP-tagged versions were expressed from their endogenous promoter sequences, at their native chromosomal location. The resulting strains were designated *CHS1–YFP*, *CHS2–YFP*, *CHS3–YFP* and *CHS8–YFP* (Table 1).

Western analysis using membrane protein preparations from the YFP-tagged strains during exponential growth confirmed that the YFP-tagged proteins were expressed in the cells (data not shown). Bands corresponding to the YFP-tagged Chs proteins were detected at a position corresponding to the predicted size of Chs proteins fused to YFP [Chs1p: 116 kDa, Chs2p: 116 kDa, Chs3p: 136 kDa, Chs8p: 125 kDa, and YFP: 25 kDa; CandidaDB (http://genolist.pasteur.fr/CandidaDB/)]. No proteins in this size range were detected in protein extracts prepared from wild-type (untagged) cells.

To assess whether the fusion proteins were functional, the remaining wild-type copy of either *CHS1* or *CHS2* was tagged with YFP in a corresponding heterozygous null mutant strain (*CHS1–YFP*/*chs1* and *CHS2–YFP*/*chs2*; Table 1). *CHS1* is an essential gene in *C. albicans* (Munro *et al*., 2001), and the *CHS1–YFP*/*chs1* strain was viable demonstrating that the Chs1p–YFP fusion protein was functional. Chs2p is responsible for the majority of the measurable chitin synthase activity in mixed membrane fraction (MMF) preparations *in vitro* (Gow *et al*., 1994; Munro *et al*., 2003). The specific activity measured in MMF prepared from the untagged heterozygous *chs2* mutant (*CHS2*/*chs2*) was approximately half that of the isogenic wild-type strain (BWP17) and there was no significant difference in the specific activity measured in MMF prepared from the untagged heterozygote (*CHS2*/*chs2*) and the tagged heterozygous mutant strains (*CHS2–YFP*/*chs2*) (specific activity expressed in nmol min^−1^ mg^−1^ for BWP17 1.16 ± 0.33; *CHS2*/*chs2* 0.60 ± 0.34; and *CHS2–YFP*/*chs2* 0.57 ± 0.10). Hence the Chs2p–YFP fusion protein was functional.

### All four YFP-tagged Chs proteins are localized at the septum just before cytokinesis in yeast cells

**Fig. 3:**
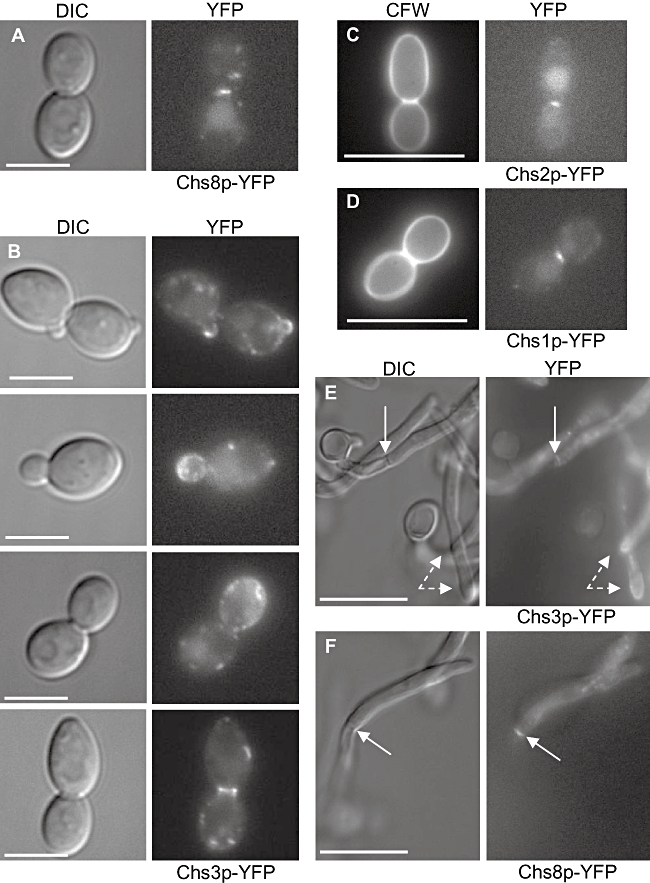
Location of the YFP-tagged Chs proteins in yeast and hyphal cells. A. Mid-log phase yeast cells of the *CHS8–YFP* strain. DIC images (left) and corresponding YFP images (right) are shown. The scale bar represents 5 μm. B. Mid-log phase yeast cells of the *CHS3–YFP* strain at different stages of the cell cycle. Left panels show DIC images and right panels show corresponding YFP fluorescence. The scale bar represents 5 μm. C and D. Mid-log phase yeast cells of the *CHS2–YFP*/*chs2* (C) and *CHS1–YFP*/*chs1* (D) strains stained with CFW (left) and corresponding YFP images (right). The scale bar represents 15 μm. E and F. Hyphal cells of the *CHS3–YFP* (E) and *CHS8–YFP* (F) grown in 20% (v/v) FCS at 37°C for 2 h. DIC images (left) and corresponding YFP images (right) are shown. Septa are indicated with solid arrows and hyphal tips with dashed arrows. The scale bar represents 15 μm.

In order to determine where the YFP-tagged Chs proteins were located during the yeast cell cycle, exponentially growing yeast cells of YFP-tagged strains were observed by fluorescence microscopy. Chs8p–YFP was localized at the mother–bud neck only when the mother and bud were approximately equal in size, presumably just before cytokinesis (Fig. 3A). Chs8p–YFP was also visible in punctate cytoplasmic patches and diffusely in vacuoles. Chs3p–YFP was located at the mother–bud neck when the mother and bud were approximately equal in size (Fig. 3B, bottom). In addition, Chs3p–YFP was localized at the tip of growing buds and in the wall of the growing bud (Fig. 3B, top), and in some small punctate patches.

We were unable to visualize Chs1p–YFP and Chs2p–YFP in exponentially growing yeast cells of the *CHS1–YFP* and *CHS2–YFP* strains; however, Chs1p–YFP and Chs2p–YFP were visible in exponentially growing yeast cells of the YFP-tagged heterozygous strains. In the *CHS2–YFP*/*chs2* strain, Chs2p–YFP was observed at the septum when mother and bud were approximately equal in size, in some punctate patches near the cell periphery and diffusely in the vacuole (Fig. 3C). In the *CHS1–YFP*/*chs1* strain, a weak signal from Chs1p–YFP was observed at the septum when the mother and bud were approximately equal in size, and in the vacuoles (Fig. 3D). No specific localization of either of these two proteins was observed at other stages in the cell cycle (data not shown). No YFP signal was detected when cells of an untagged wild-type strain were examined (data not shown). These results indicate that all four *C. albicans* chitin synthases are present at the septum in yeast cells.

### Chs8p–YFP and Chs3p–YFP are present at septa in hyphae

A similar distribution of Chs8p–YFP and Chs3p–YFP to that in yeast cells was observed when growing hyphae of the *CHS8–YFP* and *CHS3–YFP* strains were examined. Chs3p–YFP was observed at the tip of growing hyphae and at the septum (Fig. 3E) and in punctate patches which tended to be concentrated towards the tip of the growing hyphae. Chs8p–YFP was also observed at the septum (Fig. 3F) and in punctate patches close to the hyphal tip. No detectable YFP signal was observed when hyphal cells of the *CHS1–YFP*, *CHS2–YFP* and untagged wild-type strains were examined (data not shown). As Chs1p is required for the synthesis of the hyphal septum, we would predict that it would be located at the site of septum formation in hyphal cells. Our inability to visualize it at this site indicates that the signal from Chs1p–YFP (and presumably Chs2p–YFP) was below the detection limit of the camera. Therefore Chs8p and Chs3p are located at appropriate sites at an appropriate time in the cell cycle to account for the synthesis of the long- (Chs8p) and short- (Chs3p) chitin microfibrils seen in the septa and cell walls of hyphae.

## Discussion

We present evidence to suggest that specific chitin synthase enzymes are responsible for the synthesis of specific types of chitin microfibrils at different locations in the cell, and therefore the architecture of chitin at these sites of *C. albicans* is dependent on the number and types of chitin synthase enzymes present. Long-chitin microfibrils normally visible in the septum were absent when *CHS8* was deleted, and short-chitin rodlets normally visible in the cell wall were absent when *CHS3* was deleted. We therefore concluded that Chs8p is required for the synthesis of long-chitin microfibrils and that Chs3p is required for short-chitin rodlets. Visualization of Chs3p–YFP and Chs8p–YFP provided evidence that these two enzymes were present at appropriate locations at appropriate stages of the cell cycle to account for the presence of short and long microfibrils that were observed in chitin ghosts.

**Fig. 4:**
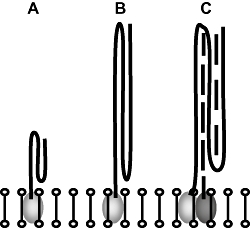
Alternative hypotheses to explain the synthesis of α-chitin microfibrils of different lengths. Individual chitin synthase enzymes may produce antiparallel chitin microfibrils of a specific length (A: short fibrils; B: long fibrils). Alternatively, two chitin synthase enzymes may cooperate in the synthesis of an individual microfibril (C) where short chains of chitin synthesized by one enzyme stabilize the nascent microfibril synthesized by another enzyme preventing folding back of antiparallel chitins hence lengthening the microfibril.

At least two non-exclusive models can be proposed to explain how α-chitin microfibrils of different lengths could be synthesized (Fig. 4). Individual chitin synthase proteins in the plasma membrane may synthesize chitin microfibrils of a particular length. For example, Chs3p may synthesize a nascent chitin chain that folds back on itself more frequently to form short rodlet-shaped microfibrils (Fig. 4A). Chs8p may synthesize less folded and hence longer-chitin microfibrils (Fig. 4B). Alternatively, two (or more) chitin synthase enzymes may cooperate in the synthesis of an individual chitin microfibril (Fig. 4C), where short-chitin oligosaccharides synthesized by one enzyme are released and then bind to and stabilize the nascent chitin chain synthesized by another, thereby preventing the nascent chain from folding back on itself hence lengthening the fibril.

Our data indicate that Chs1p, Chs2p, Chs3p and Chs8p all participate, or are present at the site of cytokinesis where the septum is synthesized in *C. albicans*. It is interesting that a difference in the microfibrillar architecture of chitin at the septum was only observed in the *chs8*Δ mutant strain, where long-chitin microfibrils were absent and short-chitin rodlets were visible. Based on our proposed models for the synthesis of chitin microfibrils of different lengths, the most simple explanation is that septal plates are comprised of a layer of long-chitin microfibrils synthesized by Chs8p that covers a layer of short-chitin rodlets synthesized by Chs3p. Alternatively, long microfibrils would also be absent in a *chs8*Δ mutant strain if Chs8p normally cooperates with other Chs enzymes to synthesize long-chitin microfibrils. The precise role of the long-chitin microfibrils in the septum remains unclear. They may act to strengthen the septum; however, *chs8*Δ mutants bud and separate normally, and the septum does not appear to be weakened as determined by the lack of buds that lyse spontaneously after cytokinesis (Munro *et al*., 2003).

Chs3p alone was visualized at the sites of polarized tip growth of buds and hyphae, which is consistent with the fact that short-chitin rodlets were predominantly observed in the cell wall of yeast and hyphal cells. It should be noted, however, that we cannot exclude the possibility that other Chs enzymes are present at these sites in low abundance. Long-chitin microfibrils were observed in the cell walls of *chs3*Δ mutant strains, and Chs1p has been shown to contribute to the stability of the cell wall (Munro *et al*., 2001). It is therefore likely that, in addition to Chs3p, other chitin synthases do, or can contribute to the synthesis of chitin in the cell wall.

We have demonstrated for the first time that individual members of a chitin synthase multigene family make specific contributions to the total chitin architecture of a fungal cell. Specific Chs proteins generate chitin microfibrils of different lengths, or may act cooperatively to generate the pattern of microfibrils observed in fungal cell walls. The implication of these observations is that chitin microfibrils of different architecture have distinct biophysical roles that may directly influence properties such as the rigidity and compliance of the cell wall.

## Experimental procedures

### Strains and media

*Candida albicans* strains used in this study are listed in Table 1. Wild-type CAF2-1 and isogenic *chs* mutant strains were grown in liquid YPD medium containing 1% yeast extract, 2% peptone, 2% glucose, and maintained on solid YPD which also contained 2% agar. Wild-type BWP17 and YFP-tagged strains were grown in YPD supplemented with uridine (YPD+Uri) at a final concentration of 25 μg ml^−1^, but were maintained on solid selective minimal medium (SD) containing 0.67% yeast nitrogen base containing ammonium sulphate, 2% glucose, 2% purified agar and appropriate auxotrophic supplements. Liquid yeast cultures were grown at 30°C with shaking at 200 r.p.m. To prepare hyphal cultures, stationary-phase yeast cells grown in YPD were washed in sterile distilled water (dH_2_O), re-suspended in 20% fetal calf serum (FCS) in dH_2_O at a concentration of 1 × 10^7^ cells ml^−1^, and incubated at 37°C with shaking at 200 r.p.m. The *chs1*Δ conditional mutant containing a single functional *CHS1* allele under the control of a *MRP1* maltose-regulated promoter (Munro *et al*., 2001) was grown in media containing 2% maltose (SMal or YPMal) to induce transcription from the promoter, and grown in glucose (SD or YPD) to repress the promoter.

### Chemical preparation of chitin ghosts

Yeast and hyphal cultures of wild-type CAF2-1 cells were grown for 6 h in 300 ml volumes of YPD+Uri or 20% (v/v) FCS as described above. Cells were harvested and cellular material other than chitin was extracted using the method described in Gow *et al*. (1980). Cells were washed with dH_2_O, boiled in 5% KOH for 30 min and washed three times with dH_2_O. The alkali-insoluble residue was re-suspended in hydrogen peroxide/glacial acetic acid (1:1) and autoclaved at 121°C for 15 min. The insoluble residue was collected by centrifugation, washed with dH_2_O three times and again boiled in 5% KOH for 30 min. Alkali-insoluble chitin from yeast cells was washed three times with dH_2_O and then stored in dH_2_O at 4°C. Prior to storage the chitinous material from hyphal cells was ultrasonicated for 1 min to fragment hyphae at septal junctions to aid the visualization of the septal plates (Gow *et al*., 1980).

### Shadow-cast TEM

Chemically or enzymatically extracted chitin ghosts were prepared for shadow-cast TEM essentially as described by Gow *et al*. (1980). Briefly, an equal volume of 0.2% bacitracin solution was added to the chitin ghosts as a wetting agent to facilitate the even coating of a formvar-coated 400 mesh copper grid. Excess liquid was removed using a Pasteur pipette leaving dispersed chitin ghosts on the grid. The grid was dried and shadowed with tungsten at an angle of 26° for 3 min in a vacuum coating unit (Edwards Coating System, E306A). The chitin microfibrils were then visualized by TEM (Philips 301 or Philips C10).

### Enzymatic preparation of chitin ghosts

Wild-type CAF2-1 yeast cells were grown for 6 h at 30°C and chitin was extracted using the method described by Kapteyn *et al*. (2000). Cells were harvested, washed in dH_2_O, re-suspended in 4% (v/v) SDS and boiled at 100°C for 3 min. The SDS-extracted cell wall material was washed with dH_2_O and treated with Glyko®β(1,6)-glucanase (ProZyme) (0.8 U per gram wet weight of cell walls) in 50 mM sodium phosphate buffer (pH 5.5) at 37°C for 18 h with shaking. The β(1,6)-glucanase-treated samples were again treated with SDS, washed in dH_2_O, and digested with Quantazyme *ylg*™ (Qbiogene) 1500 U per gram wet weight of cell walls, a recombinant β(1,3)-glucanase, at 37°C for 18 h with shaking. The samples were again washed with dH_2_O, treated with SDS, washed with dH_2_O before being prepared for examination by TEM.

### Chitinase and chitosanase treatment of chitin ghosts

Chitin ghosts were prepared by chemical means as described above. A 1 mg sample of the chitin preparation was incubated at 37°C with 0.1 U chitinase from *Streptomyces griseus* (Sigma) in 50 mM sodium phosphate buffer (pH 6.0) or 0.5 U chitosanase from *S. griseus* (Sigma) in 100 mM sodium phosphate buffer (pH 5.0). After 8 h, a second aliquot of chitinase or chitosanase was added and incubated at 37°C for a further 16 h. Samples were washed several times with dH_2_O and stored in dH_2_O.

### Construction of *C. albicans* strains

The four *C. albicans CHS* genes were fused to the gene encoding *YFP* using the method described by Gerami-Nejad *et al*. (2001). Briefly, PCR primers with 100 bp of homology to the sequence immediately upstream and downstream of the stop codons of each *CHS* gene were designed to anneal to either end of the *YFP* cassette in the plasmid pYFP-URA3 (Gerami-Nejad *et al*., 2001) (primers MDL1–MDL8; Table S1). The resulting PCR product containing *YFP*, the *ADH1* terminator sequence and the *URA3* marker gene was then transformed into *C. albicans* strain BWP17 (Table 1) using a method based on those described in Gietz *et al*. (1995) and Walther and Wendland (2003). Ura^+^ colonies were screened for the presence of the correctly integrated YFP cassette by Southern analysis using the ECL Direct Nucleic Acid Labelling and Detection System (GE Healthcare) confirming the successful creation of the strains NGY475 (*CHS1–YFP*), NGY476 (*CHS2–YFP*), NGY477 (*CHS3–YFP*) and NGY478 (*CHS8–YFP*) (Table 1). Each strain contains one wild-type copy and one 3′-YFP-tagged copy of the appropriate *CHS* gene at the chromosomal locus. To facilitate hypha formation, wild-type copies of the auxotrophic marker genes were replaced by integrating the plasmid CIp30 (Dennison, 2004) at *RPS1* after digestion with StuI.

The heterozygous strain NGY479 (*CHS2*/*chs2*; Table 1) was constructed using a PCR-based method adapted from Noble and Johnson (2005). PCR primers with 100 bp homology to the sequence immediately upstream of the start codon of *CHS2* and 100 bp immediately downstream of the stop codon of *CHS2* were designed to anneal to sequences immediately adjacent to the *Candida dubliniensis HIS1* marker (*CdHIS1*) in pSN52 (Noble and Johnson, 2005) (primers MDL20 and MDL21; Table S1). The resulting PCR product was then transformed into *C. albicans* strain BWP17. His^+^ colonies were then screened by PCR (primers MDL31 and MDL29; Table S1) to identify transformants where one copy of *CHS2* was replaced by the *CdHIS1* marker.

The wild-type copy of the *CHS* genes in the heterozygous mutant strains NGY20 (*CHS1*/*chs1*) and NGY479 (*CHS2*/*chs2*) was tagged with *YFP* and as described above resulting in the creation of the strains NGY480 (*CHS1–YFP*/*chs1*) and NGY481 (*CHS2–YFP*/*chs2*) (Table 1). As the mutant *chs1* allele in NGY20 still had some 3′ sequence of the *CHS1* ORF present, correct fusion of the *YFP* cassette to the wild-type allele of *CHS1* was confirmed by PCR (primers MDL11 and MDL16R; Table S1). As NGY479 (*CHS2*/*chs2*) contains a complete start to stop deletion of one copy of *CHS2* (*chs2*Δ*0*), the *YFP* cassette integrated at the remaining wild-type *CHS2* allele. Correct integration was confirmed by Southern analysis.

### Western analysis

Membrane proteins were prepared from 100 ml of cultures of strains BWP17 (wild-type), *CHS1–YFP*, *CHS2–YFP*, *CHS3–YFP* and *CHS8–YFP* grown in YPD+Uri for 6 h at 30°C with shaking at 200 r.p.m. Cells were harvested by centrifugation, washed in 10 ml of ice-cold dH_2_O and 1 ml of ice-cold breaking buffer [100 mM Tris-HCl pH 7.5, 0.01% (w/v) SDS, 1 mM DTT, 10% (v/v) glycerol, 1 mM EDTA] containing Complete, EDTA-free Protease Inhibitor Cocktail Tablets (Roche) before being re-suspended in 250 μl of ice-cold breaking buffer. Cells were broken in the presence of acid-washed glass beads (4 × 20 s bursts on a FastPrep machine with 1 min incubation on ice between bursts) and the lysate was cleared by centrifugation (1000 *g*, 5 min, 4°C). The supernatant was subjected to centrifugation at 10 000 *g* for 15 min at 4°C, and ultracentrifugation at 100 000 *g* for 1 h at 4°C. The resulting pellet containing membrane proteins was re-suspended in an appropriate volume of breaking buffer and the protein concentration was estimated using the method described by Bradford (1976) with BSA as a standard.

Proteins were separated by SDS-polyacrylamide gel electrophoresis (SDS-PAGE) using the XCell *SureLock*™ Mini-Cell system (Invitrogen) with NuPAGE®Novex Bis-Tris 4–12% pre-cast gels (Invitrogen) in NuPAGE® MOPS-SDS Running Buffer (Invitrogen) containing NuPAGE® Antioxidant (Invitrogen) as per the manufacturer's instructions. Fifty micrograms of protein was loaded in each lane. The proteins were transferred to Invitrolon™ PVDF Membranes (Invitrogen) in NuPAGE® Transfer Buffer containing methanol using the XCell II™ Blot Module (Invitrogen) as per the manufacturer's instructions.

Following transfer, the membranes were rinsed in tris-buffered saline (TBS) and blocked in TBS-T+5% BSA [TBS, 0.1% Tween-20, 5% (w/v) BSA] O/N at 4°C. The membranes were then incubated for 2 h at room temperature in TBS-T+2.5% BSA [TBS, 0.1% Tween-20, 5% (w/v) BSA] containing a 1:2000 dilution of Anti-GFP Antibody (Roche). The membranes were washed four times for 5 min in TBS-T (TBS, 0.1% Tween-20) and then incubated for 1 h at room temperature in TBS-T+5% BSA containing a 1:4000 dilution of Anti-mouse IgG, (Fab specific) peroxidase conjugate Antibody (Sigma). The membranes were washed four times for 5 min in TBS-T and the signal was detected using LumiGLO™ Reagent and Peroxide (Cell Signaling Technology) as per the manufacturer's instructions.

### Measurement of chitin synthase activity

Mixed membrane fractions were prepared from exponential phase yeast cells and their chitin synthase activities measured as described previously (Munro *et al*., 1998). Standard reactions for measuring chitin synthase activity were carried out in 50 μl volumes containing 50 μg of MMF protein, 25 mM GlcNAc and 1 mM UDP-GlcNAc which included 25 nCi ^14^C-labelled UDP-GlcNAc with 50 mM Tris-HCl pH 7.5 and 10 mM MgCl_2_. The reactions were incubated at 30°C for 30 min and stopped by addition of 1 ml of 66% (v/v) ethanol. The reaction mixture was filtered through GF/C filter discs (Whatman) pre-soaked in 10% (v/v) trichloroacetic acid and the reactions tubes rinsed twice with 1 ml of 1% (v/v) Triton X-100. Each filter was then washed four times with 2 ml of 66% (v/v) ethanol. The radiolabelled chitin synthesized in the above reaction was trapped on the filters and unincorporated substrate was removed by washing. Filters were dried at 80°C and their radioactivity was counted in a scintillation counter.

### Microscopy

To visualize live yeast cells, the *CHS–YFP* strains were grown in liquid YPD+Uri for 4 h at 30°C with shaking at 200 r.p.m. Samples were harvested, washed in phosphate-buffered saline (PBS) and mounted on a slide under a coverslip. In order to visualize growing hyphae, overnight cultures grown in YPD+Uri were diluted 1:50 into 20% (v/v) FCS in dH_2_O pre-warmed to 37°C and incubated for 2 h at 37°C with shaking at 200 r.p.m. Samples were placed on a microscope slide at 37°C and allowed to settle for 5 min. The liquid was aspirated and cells covered with 5 μl of a molten mixture containing 20% (v/v) FCS in 1% (w/v) low-melt agarose in dH_2_O. A coverslip was applied immediately and hypha were visualized in a constant temperature hood surrounding the microscope maintained at 37°C. The *CHS–YFP* strains were examined using a DeltaVision RT microscope (Applied Precision) equipped with a CoolSNAP camera (Photometrics). YFP fluorescence was detected using a YFP filter set (Chroma).

The YFP-tagged heterozygous strains (*CHS–YFP*/*chs*) were grown in liquid YPD+Uri for 4 h at 30°C with shaking at 200 r.p.m. Samples were harvested, washed in PBS and mounted on a slide under a coverslip with 1 μl of a 10 μg ml^−1^ solution of Calcofluor White (CFW). The strains were examined using a DeltaVision RT microscope (Applied Precision) equipped with a QuantEM:512SC camera (Photometrics). YFP fluorescence was detected using a standard FITC filter set (Chroma). CFW stain was detected using a standard DAPI filter set (Chroma).

## Supplementary material

This material is available as part of the online article from: http://www.blackwell-synergy.com/doi/abs/10.1111/j.1365-2958.2007.05990.x

(This link will take you to the article abstract).
