# Supplementary material for: Individual chitin synthase enzymes synthesize microfibrils of differing structure at specific locations in the Candida albicans cell wall
Source: Mol Microbiol. 2007 Dec;66(5):1164–73. doi: 10.1111/j.1365-2958.2007.05990.x (PMC2780561; doi:10.1111/j.1365-2958.2007.05990.x)
Supplement: Supplementary file 1 [file mmi0066-1164-SD1.pdf]

Supplementary Material:

Individual chitin synthase enzymes synthesize microfibrils of differing structure at specific locations in the *Candida albicans* cell wall

Megan D. Lenardon[1,3], Rhian K. Whitton[1,3], Carol A. Munro[1], Deborah Marshall[2] and Neil A. R. Gow[1,4].

[1]School of Medical Sciences, University of Aberdeen, Institute of Medical Sciences, Foresterhill, Aberdeen AB25 2ZD, UK. [2]Department of Medical Microbiology, University of Aberdeen, Polworth Building, Foresterhill, Aberdeen AB25 2ZD, UK.

[3]These authors contributed equally to this work
[4]Corresponding author: School of Medical Sciences, University of Aberdeen, Institute of Medical Sciences, Foresterhill, Aberdeen AB25 2ZD, UK. Phone: +44 1224 555 879; Fax: +44 1224 555 844; email: n.gow@abdn.ac.uk

Table S1: Oligonucleotides used in this study.

Name	Use	Sequence (5′-3′)
MDL1	C-terminal tag of *CHS1*	TCTTGGATACTTGATACAAACATATGCACGGTTTTTGTGGA ATCGAAGAGTAAATGGATGAAACGAGGATATACCGCGCCGA GTCACAATCCATTAAATGGTGGTGGTTCTAAAGGTGAAGAA TTATT
MDL2	C-terminal tag of *CHS1*	GTTTGTACAAATTACACTACTCCTTCGCCCTTCTTTTTCTCTA ATTGATTATTAATTCTAAACACCTATTTAGCTCTAACATCAC CACGGTCTTCATTTTTCTAGAAGGACCACCTTTGATTG
MDL3	C-terminal tag of *CHS2*	GACAGTGGCATTTATGGCATTATTTAGATTTATTGGTTGTAT TTATTATTTGATCACTAGATTGGGTAGAGAAATTAAGGCAAG TGAACATGCCACTAAAGGTGGTGGTTCTAAAGGTGAAGAAT TATT
MDL4	C-terminal tag of *CHS2*	TTATACTACAGGACTCTAATCTCATTTCTTCTAAACTATAAC AACAAAATACAATTTCTCAATATACAAATATAAATATAAAT ATAATTATATATATCTATCTAGAAGGACCACCTTTGATTG
MDL5	C-terminal tag of *CHS3*	TAGTAATAATTTGGCTGTTCCTGGTGCTGCTTGGGATCCATC TAATACTGGGGGAAATTTGATTGATGATTTAAGTCAAGGATC TTCTTCAGGGTCCAGTGGTGGTGGTTCTAAAGGTGAAGAATT ATT
MDL6	C-terminal tag of *CHS3*	GTAAATGACGCAAAAATATAATCTGTCTTTTTTATATTGTTA ATAATTTTATATAACCATATACATAAATAAAAGTCCTTTCTC TCTCTCTTTTTTAAGCTCTAGAAGGACCACCTTTGATTG
MDL7	C-terminal tag of *CHS8*	AGCATTTTTATTCTTGATATTCAAGAGTTTCCGTCCACTTAAA TGGAAATTGAGAGCTAAACGAGAAAACAAACGTAATAGAA ATCAAAATAGAAATGTCGGTGGTGGTTCTAAAGGTGAAGAA TTATT
MDL8	C-terminal tag of *CHS8*	TATATTTCTAATACTGTATACCCCCTCCCAGATAAAACCAAA TTGAAAAAAAAGAAACATTTAATATAAATCTATATGTACA GAAAGGTCTCAAATATATCTAGAAGGACCACCTTTGATTG
MDL16R	YFP Southern probe (R) and screening primer in YFP ORF	CATACCATGGGTAATACCAG
MDL17	YFP Southern probe (F)	GGTTGAATTAGATGGTGATG
MDL20	Deletion of complete *CHS2* ORF	AGGTTAAACAAGGATTTCATTTTTATTCCCAACTACTTTCAC TTTCACTTTCACTTTCAAGGTCAGGAGTGGTTTTTATATATAC AACAACTAAACAAAAGCTCGGATCCACTAGTAACG
MDL21	Deletion of complete *CHS2* ORF	GACTCTAATCTCATTTCTTCTAAACTATAACAACAAAATACA ATTTCTCAATATACAAATATAAATATAAATATAATTATATAT ATCTAAAAATATCAT TCCAGTGTGATGGATATCTGC
MDL31	Screening primer upstream of *CHS2* ORF	GCTTGGATTAAATTCTTCTTAGTCG
MDL29	Screening primer in *CdHIS1* ORF	CCATTTGGCTCTTCACTACC
MDL11	Screening primer in *CHS1* ORF	CAGGTTCATTGGTGTCTTAC

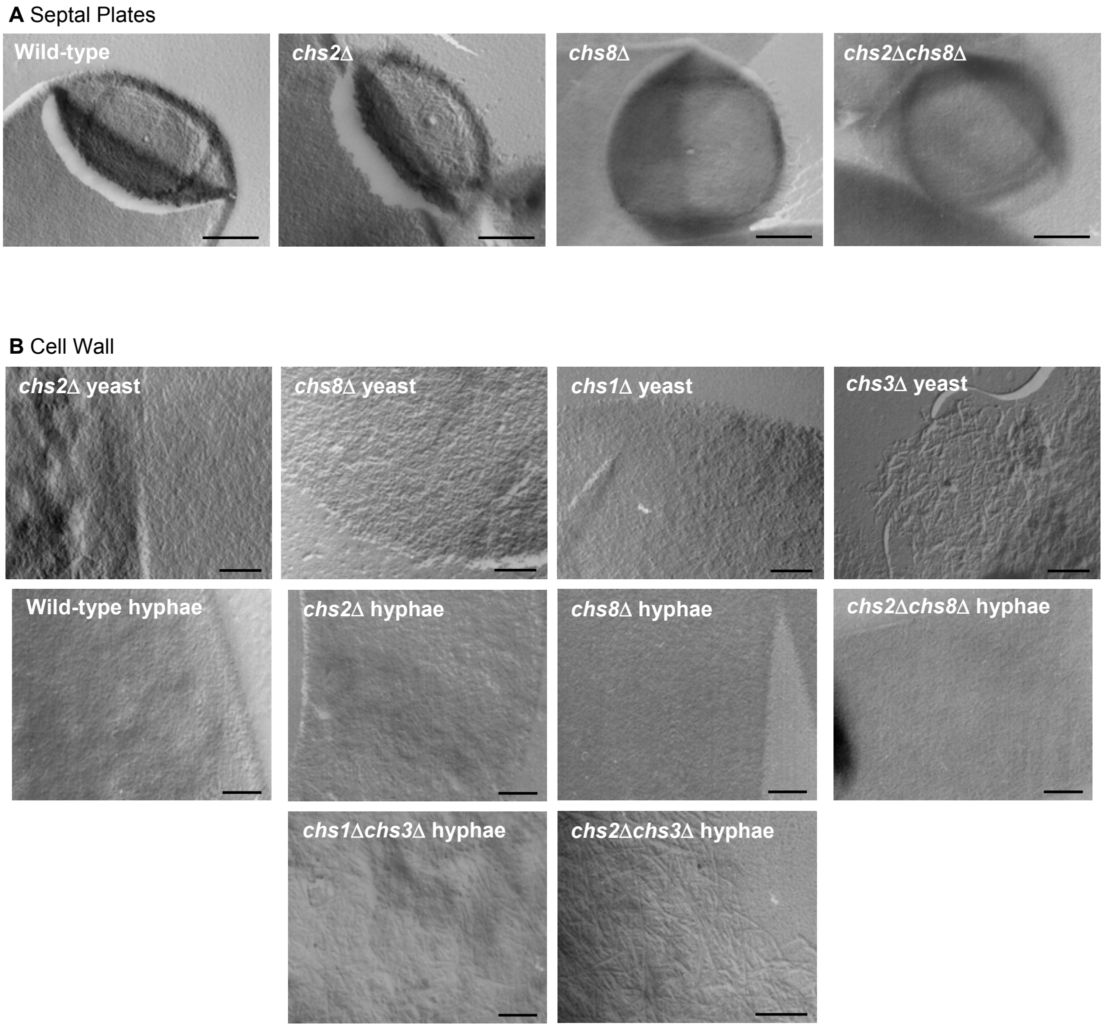

Figure S1: Microfibrillar architecture of chitin ghosts of *chs* mutant strains. A: Chitin ghosts of septal plates of hyphal cells imaged by shadow-cast TEM. The long chitin microfibrils observed in the wild-type and *chs2*Δ mutant strain are clearly absent in the *chs8*Δ and *chs2*Δ*chs8*Δ mutant strains. **B:** Chitin ghosts of cell walls of yeast and hyphal cells as indicated on the diagram. The short chitin rodlets observed in the cell wall of the wild-type, *chs2*Δ, *chs8*Δ, *chs2*Δ*chs8*Δ and *chs1*Δ mutant strains are absent in the *chs3*Δ, *chs1*Δ*chs3*Δ and *chs2*Δ*chs3*Δ strains. The scale bars represent 0.5 μm.
